# Reverse shock index (RSI) as a predictor of post-intubation cardiac arrest (PICA)

**DOI:** 10.1186/s12245-023-00569-y

**Published:** 2023-12-07

**Authors:** Mehdi Torabi, Ghazal Soleimani Mahani, Moghaddameh Mirzaee

**Affiliations:** 1 Department of Emergency Medicine, Emergency Medicine, Kerman University of Medical Sciences, Kerman, Iran; 2 Faculty of Medicine, Kerman University of Medical Sciences, Kerman, Iran; 3 Biostatistics, Modeling in Health Research Center, Institute for Futures Studies in Health, Kerman University of Medical Sciences, Kerman, Iran

**Keywords:** Cardiac arrest, Emergency department, Intubation, Vital signs

## Abstract

**Background:**

Endotracheal intubation (ETI) in critically ill patients is a high-risk procedure due to the increased risk of cardiac arrest, and several factors may predict poor outcomes in these patients. The aim of this study was to investigate the role of some factors, especially newly introduced vital signs such as the reverse shock index (RSI), in predicting post-intubation cardiac arrest (PICA) in critically ill adult patients.

**Methods:**

This cross-sectional study was conducted on critically ill patients over 18 years of age who were admitted to the emergency department (ED) and underwent ETI within 1 year. Patients who developed PICA and those without this event were included in the study, and their features were compared. The primary outcome was cardiac arrest.

**Results:**

Of 394 patients, 127 patients were included, of whom 95 (74.8%) developed PICA, and 32 (25.2%) did not experience cardiac arrest after intubation. In multivariate analysis, age, RSI, oxygen saturation, and total bilirubin were significantly associated with PICA. In addition, patients with RSI < 1 had a significantly higher risk of developing PICA (odds ratio = 5.22, 95% CI 1.83–14.86, *p* = 0.002). The sensitivity, specificity, positive predictive value, negative predictive value, and diagnostic accuracy for predicting PICA were 51.11%, 83.33%, 90.2%, 36.23%, and 59.17%, respectively. The ROC curve for RSI showed an area under the curve (AUC) of 0.66.

**Conclusion:**

RSI may be useful in predicting PICA with higher diagnostic accuracy compared to the shock index. Furthermore, advanced age, hypoxia, and hyperbilirubinemia may increase the risk of PICA in patients admitted to the ED.

## Introduction

Many critically ill patients referred to the emergency department (ED) develop complications during endotracheal intubation (ETI). In addition to the complications caused by unsuccessful intubation, one of the recognized consequences of ETI is cardiac arrest [1]. Cardiac arrest following emergent ETI in critically ill patients can be a life-threatening complication rendering a poor prognosis for these patients. This complication usually occurs within the first minutes after intubation [2]. Therefore, ETI is a risky procedure in critically ill patients, and post-intubation cardiac arrest (PICA) is a significant contributor to the high mortality rate and poor outcomes of intubated patients compared to non-intubated patients [3, 4].

Several factors may be involved in the occurrence of cardiac arrest during ETI. Several studies have outlined the role of factors such as obesity, age over 75 years, hypotension, hypoxemia, pre-intubation metabolic acidosis, the number of tracheal intubation attempts, and the use of vasopressors and neuromuscular block agents prior to intubation in propensity to cardiac arrest during ETI [5–7]. Moreover, in critically ill patients, vital signs such as heart rate, blood pressure, and shock index can be helpful in determining disease severity and prognosis [8].

In addition to these vital signs, one of the shock indices that can help predict the prognosis of critically ill patients is the Reverse Shock Index (RSI). This index is defined as the ratio of systolic blood pressure to heart rate and may be a predictor of outcomes in critical conditions. The RSI can be easily calculated using readily available vital signs obtained at the time of emergency department admission and can be used to predict prognosis in critically ill patients. Mustafa İçer et al. showed that shock indices such as RSI have diagnostic value in predicting mortality in burn patients [9]. GC Oh et al. in their study showed that in patients hospitalized for heart failure, RSI could be used as a tool to assess patient status and guide physicians in the management of patients with heart failure [10]. Jung-Fang Chuang et al. mentioned in their study that the concept of RSI is particularly valuable in crowded EDs for identifying high-risk patients and can serve as a principal trigger for action in the ED to alert trauma surgeons to the need for early intervention and timely preparation upon patient arrival, especially for those patients who are triaged [11]. W–H Lai et al. also showed that this index may be helpful in predicting in-hospital mortality in trauma patients with poorer prognoses. A decrease in this index below 1 may predict poor outcomes in high-risk patients, which can help identify patients with poor prognoses as early as possible and better prioritize patients with critical conditions during hospital triage. In addition, this index, along with other clinical signs, may play a critical role in determining the hemodynamic status of patients [12].

Although cardiac arrest following ETI is dangerous and associated with a poor prognosis, it can be potentially preventable. The RSI is a newly introduced vital sign that has not been extensively studied in the context of PICA. The present study’s aim was to investigate the role of deterministic factors, particularly newly introduced vital signs such as the RSI, in predicting PICA in critically ill adult patients referred to the ED in order to find solutions to reduce the occurrence of this event in these patients.

## Method

### Study design

This cross-sectional study was conducted on critically ill patients referred to the emergency department of Afzalipour Academic Educational Hospital. This hospital is the main referral center for internal medicine with bed number 450 in Kerman, a large city with a population of nearly 1 million in southeastern Iran, for 1 year from April 1, 2022, to April 1, 2023. The annual ED census for internal medicine patients—who constitute our study population—is more than 45,000 in Afzalipour Hospital.

All non-traumatic critically ill patients over the age of 18 who were referred to the ED and underwent ETI were included in the study. In the ED, the decision to use ETI was made by emergency medicine residents (PGY-2,3) or specialists who had successfully completed advanced cardiac life support courses, based on the patient's clinical status and the need for assisted ventilation to maintain oxygenation and ventilation. Other inclusion criteria for intubation were the need for airway protection, respiratory failure, apnea or impending respiratory arrest, upper airway obstruction, acute heart failure, and shock. Rapid sequence intubation (RSI) was a specialized procedure for endotracheal intubation used in our emergency department. The drugs used in RSI were as follows: lidocaine, fentanyl, and succinylcholine as a fixed component along with induction agents (Etomdate, propofol, etomidate) prescribed based on the physician's decision. Exclusion criteria were trauma patients under 18 years of age, pregnancy, cardiac arrest prior to ETI, pre-hospital ETI, ETI in another hospital, and lack of adequate documentation.

Cardiac arrest was defined as the absence of a pulse in combination with one of the following: ventricular fibrillation, ventricular tachycardia, asystole, and pulseless electrical activity (PEA). Then these patients were divided into two groups with or without PICA. The features of these patients were compared between the two groups. Patient information was collected from medical records and recorded in a pre-prepared checklist by a medical intern.

### Ethics committee approval

The present study was approved by the Research Ethics Committee of Kerman University of Medical Sciences under the ethics code of IR.KMU.AH.REC.1402.020. The collected data remained confidential with the researchers and were used for research purposes only.

### Statistical analysis

The data collected were analyzed by SPSS version 26 software at a significance level of *P* < 0.05. Qualitative data were described as numbers and percentages, and quantitative variables were presented as mean ± standard deviation. The association of the variables studied with the risk of PICA was ascertained by calculating the odds ratio and 95% confidence interval. Also, the *t*-test, ANOVA, and chi-square or Fisher’s exact tests, as well as logistic regression were used to compare variables between groups and determine the relationship between target variables and cardiac arrest. Initially, univariate logistic regression was performed to identify variables with a significant statistical association with PICA. Then, variables with *p* < 0.25 in the univariate model were entered into the multivariate model [13]. Finally, ROC curve analysis was conducted for the variables showing a statistically significant relationship with the outcome.

## Results

### Basic characteristics

**Fig. 1:**
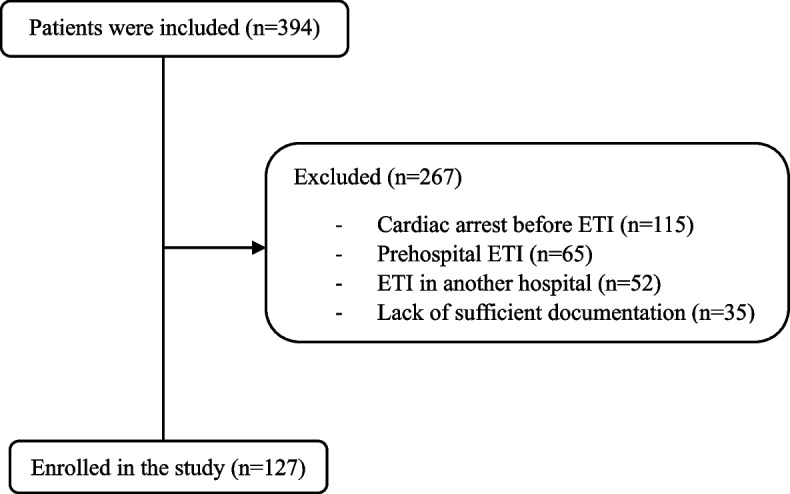
Flow chart showing enrollment of patients

Out of 394 patients, those who suffered from cardiac arrest before ETI (*n* = 115), patients who were intubated by the staff of emergency medicine service (EMS) (*n* = 65), those who were referred to our hospital after intubation (*n* = 52), and patients whose information was incomplete (*n* = 35) were excluded from the study. Finally, 127 individuals were included in the study. Of whom 95 (74.8%) suffered from PICA, and 32 (25.2%) had no cardiac arrest after intubation (Fig. 1).

Of these, 77 (60.63%) and 50 (39.37%) patients were men and women, respectively. Then the features of patients with and without PICA were compared. Cardiac arrest was considered the primary outcome in the patients. General characteristics of the patients have been shown in Table 1 (categorical and quantitative variables).

**Table 1:** Patient’s characteristics for qualitative and quantitative variables according to their association with PICA

Variables	Cardiac arrest		*p* value
	Yes	No	
Sex			
Female	45 (47.9%)	5(15.6%)	0.002
Male	50(52.1%)	27(84.4%)	
PMH^a^			
DM^b^	21(22.8%)	3(9.4%)	0.09
HTN^c^	28(30.4%)	3(9.4%)	0.02
IHD^d^	12(13.0%)	2(6.3%)	0.51
CVA^e^	11(12.0%)	2(6.3%)	0.51
CKD^f^	5 (5.4%)	1(3.1%)	1.00
COPD^g^	26(28.3%)	3(9.4%)	0.03
Malignancy	18(19.6%)	1(3.1%)	0.02
Cirrhosis	4(4.4%)	0(0%)	1.00
HIV	1(1.1%)	0(0%)	1.00
ECG			
Rate changes			
ST^h^	64(68.1%)	12(37.5%)	0.007
SB^i^	3(3.2%)	1(3.1%)	
Blocks			
RBBB^j^	10(10.6%)	3(9.4%)	0.70
LBBB^k^	5(5.3%)	0(0%)	
Bifascicular	3(3.3%)	0(0%)	
ST-T changes	5(5.4%)	0(0%)	1.00
Crystalloid before ETI^l^	54(56.3%)	22(68.8%)	0.21
Vasopressor before ETI	26(27.1%)	3(9.4%)	0.03
Oxygenation type			0.003
Non-rebreathing mask	74(78.6%)	13(43.4%)	
Simple mask	14(14.8%)	12(40.1%)	
Nasal cannula	1(1.1%)	2(6.6%)	
Venturi mask	2(2.2%)	2(6.6%)	
BiPAP^m^	3(3.3%)	1(3.3%)	
Intubation indications			
Airway protection	66(68.8%)	28(87.5%)	0.03
Respiratory failure	49(51.0%)	7(21.9%)	0.004
Acute heart failure	2(2.1%)	0(0%)	1.00
Septic shock	29(30.2%)	1(3.1%)	0.002
Cardiogenic shock	7(7.4%)	0(0%)	0.2
Obstructive shock	7(7.4%)	0(0%)	0.2
Anaphylactic shock	0(%)	1(3.1%)	0.25
Induction agents			0.005
Etomidate	52(54.7%)	19(59.4%)	
Propofol	10(10.5%)	10(31.3%)	
Ketamine	2(2.1%)	0(0%)	
NMBA^n^			
Succinylcholine	8(8.5%)	6(18.8%)	0.2
Intubation in first try	11(11.5%)	1(3.1%)	0.3
Age, mean ± SD	65.53 ± 19.37	38.22 ± 18.19	< 0.0001
Vital signs			
PR^o^, mean ± SD	113.62 ± 27.93	101.25 ± 22.11	0.01
SBP^p^, mean ± SD	113.7330.07	132.7524.93	0.02
MAP^q^, mean ± SD	84.52 ± 22.51	105.41 ± 20.82	0.02
SI^r^, mean ± SD	1.07 ± 0.38	0.78 ± 0.23	0.001
RSI^s^, mean ± SD	1.06 ± 0.39	1.36 ± 0.44	0.005
O2 saturation, mean ± SD	82.35 ± 15.38	84.00 ± 16.08	0.006
Lab tests			
Hb, mean ± SD	12.62 ± 3.25	13.65 ± 2.25	0.001
BUN, mean ± SD	80.08 ± 41.53	32.25 ± 9.74	< 0.0001
Cr, mean ± SD	1.87 ± 1.60	0.95 ± 0.33	0.008
Bili T, mean ± SD	3.26 ± 4.06	0.75 ± 0.30	0.002
Bili D, mean ± SD	2.01 ± 3.06	0.25 ± 0.10	0.005
AST, mean ± SD	263.31 ± 593.78	67.75 ± 74.33	0.01
ALT, mean ± SD	118.69 ± 131.12	44.50 ± 24.55	0.02
ESR, mean ± SD	48.62 ± 44.59	17.00 ± 14.78	0.02
CRP, mean ± SD	105.26 ± 104.58	20.45 ± 23. 05	0.06

^a^Past medical history

^b^Diabetes mellitus

^c^Hypertension

^d^Ischemic heart disease

^e^Cerebrovascular accident

^f^Chronic kidney disease

^g^Chronic obstructive pulmonary disease

^h^Sinus tachycardia

^i^Sinus bradycardia

^j^Right bundle branch block

^k^Left bundle branch block

^l^Endotracheal intubation

^m^Bi-level positive airway pressure

^n^Neuromuscular block agent

^o^Pulse rate

^p^Systolic blood pressure

^q^Mean arterial pressure

^r^Shock index

^s^Reverse shock index

### Univariate analysis

All variables with a potentially significant relationship with cardiac arrest as an outcome were investigated in univariate analysis to determine if they could retain a statistically significant association. Age, pre-intubation vital signs, hemoglobin, BUN, Cr, bilirubin, AST, ALT, ESR, and CRP, as well as gender, underlying diseases (hypertension, COPD, and malignancy), heart rate changes, receiving vasopressor prior to ETI, type of oxygenation received, indications for ETI (airway protection, respiratory failure, septic shock), the medications received for induction were found to be the variables independently associated with PICA (Table 1). These variables were further analyzed to measure association with outcome by calculating odds ratios (OR) and 95% confidence interval (95% CI) to estimate the precision of the OR in this analysis.

### Multivariate analysis

In the multivariate model, four variables showed a significant association with PICA, including age, RSI, oxygen saturation, and total bilirubin level (Table 2). There was a significant relationship between an RSI of < 1 [11] and the risk of PICA (OR = 5.22, 95% CI 1.83–14.86, *p* = 0.002), meaning that for each unit decrease in RSI, the chance of PICA would increase by 5.22 times.

**Table 2:** Univariate/multivariate regression analysis of variables according to their association with PICA

Variables	Univariate logistic regression			Multivariate logistic regression		
	Odds Ratio	95% CI	*p* value	Odds ratio	95% CI	*p* value
Age	1.06	1.04–1.09	< 0.0001	1.06	1.03–1.1	< 0.0001
Sex						–
Female Male	4.96 ref	1.76–13.98	0.002	–	–	
PR^a^	1.02	1.00–1.03	0.01	–	–	–
SBP^b^	0.98	0.96–0.99	0.02	–	–	–
MAP^c^	0.97	0.95–0.99	0.02	–	–	–
SI^d^	15.31	2.93–79.86	0.001	–	–	–
RSI^5^	0.25	0.09–0.65	0.005	0.20	0.060.63	0.006
O2 saturation	0.94	0.90–0.98	0.006	0.95	0.90–0.99	0.03
Hb	0.77	0.67–0.90	0.001	–	–	–
BUN	1.04	1.02–1.06	< 0.0001	–	–	–
Cr	2.37	1.25–4.46	0.008	–	–	–
Bili T	4.18	1.72–10.19	0.002	3.44	1.22–9.71	0.01
Bili D	13.19	2.15–80.99	0.005	–	–	–
AST	1.01	1.00–1.02	0.01	–	–	–
ALT	1.01	1.00–1.03	0.02	–	–	–
ESR	1.02	1.00–1.04	0.02	–	–	–
CRP	1.03	0.99–1.06	0.06	–	–	–

^a^Pulse rate

^b^Systolic blood pressure

^c^Mean arterial pressure

^d^Shock index

^e^Reverse shock index

### ROC curve

**Fig. 2:**
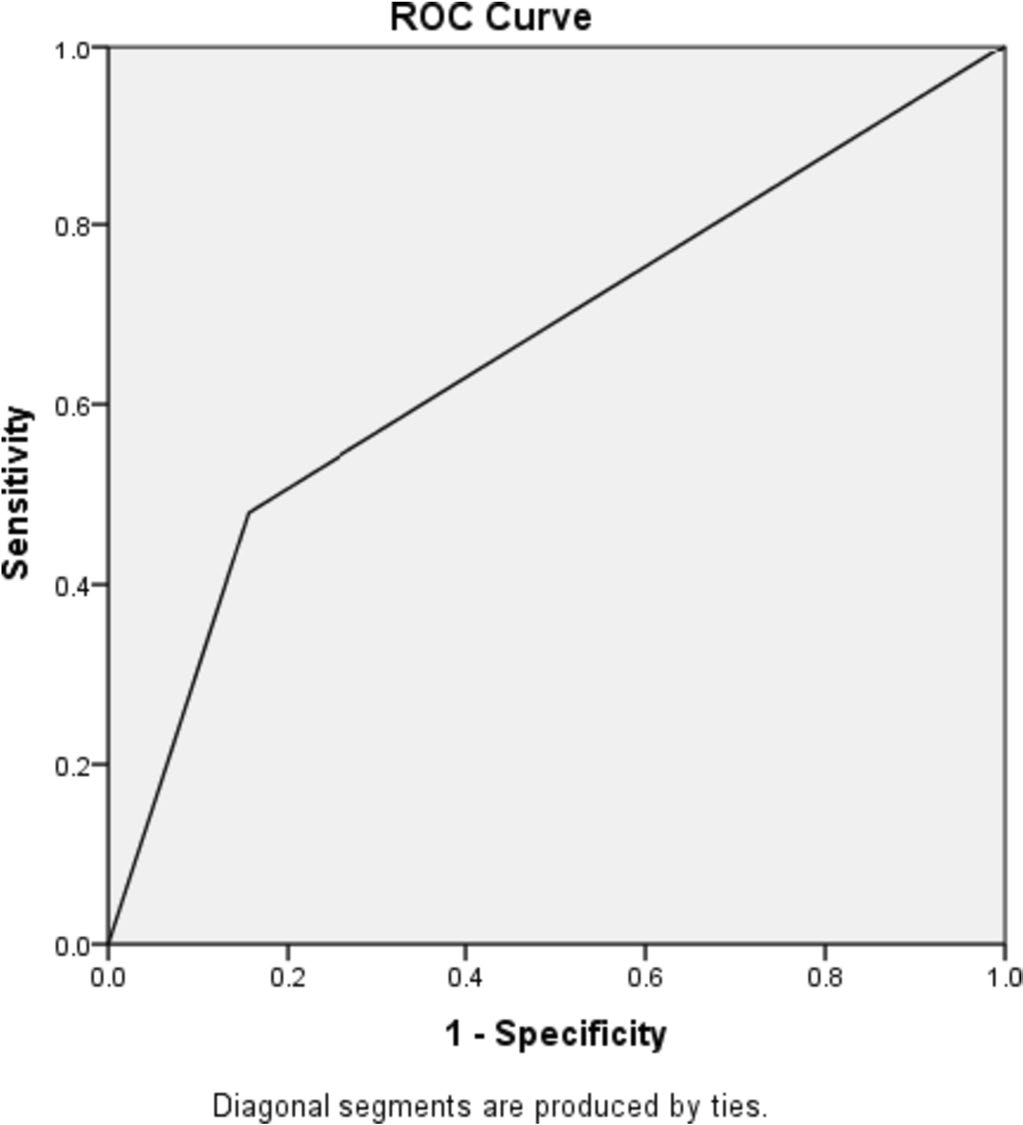
Receiver operating characteristic curve for RSI in predicting PICA

The receiver operating characteristic (ROC) curve was drawn for RSI, and the area under the curve (AUC) was calculated as 0.66 (Fig. 2).

For RSI values less than < 1, sensitivity, specificity, positive predictive value, negative predictive value, and diagnostic accuracy for predicting PICA in the patients referred to the ED were obtained as 51.11%, 83.33%, 90.2%, 36.23%, and 59.17%, respectively. The positive and negative likelihood ratios for predicting PICA were calculated as 3.06 and 0.58, respectively (Table 3).

**Table 3:** AUC, sensitivity, specificity, PPV, NPV, LR, and accuracy of RSI for predicting PICA

	AUC^a^ ^(95%CI)^	Sensitivity ^(95%CI)^	Specificity ^(95%CI)^	PPV^b^ ^(95%CI)^	NPV^c^ ^(95%CI)^	LR^d^( +) ^(95%CI)^	LR ( −) ^(95%CI)^	Accuracy ^(95%CI)^
RSI^e^	0.66	51.11% (40.95–61.18)	83.33% (66.44–92.66)	90.2% (79.02–95.74)	36.23% (25.90–48.02)	3.06 (1.98–4.72)	0.58 (0.55–0.62)	59.17% (50.22–67.54)

^a^Area under curve

^b^Positive predictive value

^c^Negative predictive value

^d^Likelihood ratio

^e^Reverse shock index

## Discussion

Several factors can help emergency medicine physicians predict PICA in the ED, among which vital signs because they can be easily calculated and monitored, play a significant role in physicians’ decision-making in critical situations. One of the newly introduced vital signs is the RSI. Our study showed that the RSI may be able to play a significant role in predicting PICA in critically ill patients in the ED. Other potential predictors included advanced age, hypoxia, and hyperbilirubinemia.

Regarding the relatively high prevalence of PICA and its association with a high mortality rate in critically ill patients, it is important to avoid ETI as much as possible. In this condition, the hemodynamic status and vital signs of patients before intubation can play a key role in predicting upcoming PICA [14–17]. Side effects occur in more than 30% of patients undergoing ETI, and the incidence of this condition is more common in patients with unstable hemodynamics. Therefore, all emergency medicine physicians should think ahead and plan properly for hypoxia, hypotension, and cardiac arrest during ETI considering that circulatory collapse during ETI can be a result of hypoxemia and shock. Several studies have confirmed the role of hemodynamic factors such as hypoxemia, hypotension, and shock index in predicting cardiac arrest following intubation [18, 19].

Hypoxia and hypotension are among the factors that can increase the risk of PICA in critically ill patients. Patients with hypotension prior to intubation have a higher probability of developing PICA than individuals who are hemodynamically stable. In a study by Russell et al., it was confirmed. They also showed that intravenous bolus administration of crystalloids in critically ill patients could not prevent cardiovascular collapse in these patients [20]. Russotto et al. declared a prevalence of 43.4% for hemodynamic disturbance during ETI and reported a higher incidence of PICA in patients with hypoxemia, hypotension, and tachycardia. Similar to the report of Russell et al. bolus administration of crystalloids and even vasopressors did not considerably reduce the incidence of cardiac arrest after ETI [21]. In their study, VanDeWall et al. showed that the probability of PICA was higher in high-risk patients, including those suffering from hypotension and persistent hypoxemia [22].

The shock index is another hemodynamic status, which is defined as the ratio of heart rate to systolic blood pressure. A significant relationship has been reported between an elevated shock index and PICA. When the shock index exceeds 0.9, the risk of cardiac arrest after intubation increases significantly [23, 24]. Torabi et al. in a study on patients with level II triage, showed that the shock index had a considerably higher value than hypotension in predicting hospital mortality in non-traumatic patients and could better identify patients with critical conditions compared to blood pressure [25]. In the present study, hypoxia, hypotension, and shock index were identified to be important predictors of PICA; however, these factors, with the exception of hypoxia, did not retain their significant association in the multivariable model.

In addition to the shock index, the RSI can also be efficient in assessing the hemodynamic status of patients. When the RSI falls below 1, it can indicate the unstable condition of patients referred to the ED even those without signs of hypotension [26]. The RSI, similar to the shock index, can predict not only hospital mortality but also other outcomes with a poor prognosis. These two indices have a significant correlation with adverse prognosis in patients [27]. Most of the studies conducted on the RSI have addressed the applicability of this index along with the Glasgow Coma Scale (GCS) (i.e., RSI*GCS) as a reliable indicator for predicting in-hospital mortality in trauma patients in hospital triage. In a study, Shao-Chun Wo et al. showed that the RIS had a higher predictive accuracy for in-hospital mortality in trauma patients compared to the shock index [28]. In another study, Po-Chen Lin et al. affirmed the superiority and higher accuracy of the RSI in predicting mortality in patients with traumatic head injuries compared to the shock index [29]. Likewise, Chen et al. emphasized that the RSI attained a higher diagnostic accuracy than the shock index in determining the poor functional outcome of trauma patients [30]. Anyway, the decision to intubate critically ill patients in the emergency department has always been a two-way street, as PICA has been common and sometimes troublesome for physicians. As you can see in this study, the importance of vital signs has been emphasized. Along with the old vital signs, we pointed out the importance of the shock indices, in the meantime, the result of the study pointed out the significant role of the reverse shock index in predicting PICA and this role was somehow more important than the shock index. In the univariate model, there was a significant relationship between PICA and the shock index as well as the reverse shock index, but in the multivariate model, this relationship was only significant with the reverse shock index. In the present study, among the vital signs assessed, only the reverse shock index and oxygen saturation remained independent predictors of PICA in the multivariable model.

Age is another factor that can be associated with poor outcomes after ETI in patients referred to the ED. With advanced age, the risk of cardiac arrest after ETI is intensified in patients. In a study by Colleran et al. over 50% of patients developed cardiac arrest following intubation, among whom older patients showed a poorer prognosis [31]. Consistent with our observation in the present study, several other studies have also pointed out that older patients carry a higher risk of hypotension and cardiac arrest after intubation [7, 17, 21].

Serum bilirubin concentration is often used to assess liver function in critically ill patients, and hyperbilirubinemia is an indicator of liver dysfunction. Pierrakos et al. investigated hyperbilirubinemia in critically ill patients and reported that it was an independent predictor of mortality in these patients [32]. In another study, Patel et al. investigated the association of serum bilirubin levels with the outcome of patients with sepsis and demonstrated that elevated levels of serum bilirubin in the first 72 h of hospitalization predicted a higher rate of in-hospital mortality in patients with severe sepsis and septic shock [33]. In another study searching for mortality predictors in critically ill hospitalized patients, it was identified that total serum bilirubin level was independently associated with in-hospital mortality. Yang et al. declared that hyperbilirubinemia, along with hypoxia, advanced age, tachycardia, and hypotension, could be helpful in predicting in-hospital mortality in critically ill patients [34]. In the present study, we observed that total hyperbilirubinemia can be a predictor of PICA in patients referred to the ED.

## Limitations

This study has some limitations. First, it had a retrospective design, so we had to exclude patients with incomplete data profiles. Second, this was a single-center study conducted in the internal medicine center for adults with internal diseases, so children, trauma patients, and pregnant women were not included. Also, patients who developed cardiac arrest before intubation and those whose ETI was performed in other centers were excluded from the study.

## Conclusion

The reverse shock index (RSI) may be able to predict PICA with higher diagnostic accuracy than the Shock Index in adult patients presenting to the emergency department. In addition, advanced age, hypoxia, and hyperbilirubinemia may predict a higher risk of PICA in these patients. It is recommended to conduct more studies with larger sample sizes in this area.

## Data Availability

Not applicable.
